# Nevus Lipomatosus Cutaneous Superficialis: A Rare Case Report

**Published:** 2014-05

**Authors:** Sunita B Patil, Shilpa Narchal, Madhura Paricharak, SS More

**Affiliations:** Department of Pathology, D.Y. Patil Medical College and Research Centre, Kolhapur, India

**Keywords:** Hamartoma, Nodule, Differential diagnosis

## Abstract

Nevus lipomatosus cutaneous superficialis is a rare idiopathic hamartomatous anomaly of the skin characterized by the presence of clusters of mature fat cells among the collagen bundles of the dermis. The classic, or solitary type, presents with asymptomatic soft, skin-colored to yellow papules or nodules. We report the case of a 12-year-old boy with congenital, classical nevus lipomatosus cutaneous superficialis that started as a single painless nodule. Over time, the nodule developed into lesions that presented as skin-colored, well-defined, soft sessile growths with a cerebriform surface centered by comedo-like plugs that increased in size and spread over a large area (approximately 12×4 cm) over the right gluteal region. We report this case as it is an uncommon condition with the intent to highlight its clinical and histopathological features, and differential diagnosis.

## Introduction

Nevus lipomatosus cutaneous superficialis (NLCS) of Hoffmann-Zurhelle is an uncommon cutaneous hamartoma characterized by the presence of mature adipose tissue in the dermis.^1^ It is also known as pedunculated lipofibroma. Comprised of fat, this nevoid lesion is present in papillary and reticular layers of the dermis.^2^^,^^3^ Clinically it is classified as either a classical type (multiple) or solitary type.^1^ “Groups of multiple, soft, non-tender, pedunculated or sessile, cerebriform, yellowish or skin colored papules, nodules or plaques” define the classical form.^4^ Classical lesions are usually present at birth or in the first two to three decades of life in a zonal or segmental distribution over the buttocks, lower back or upper thighs whereas solitary types develop in adults and show a wider distribution in the skin.^1^^,^^4^Both types show similar histological features, with varying proportion of adipose tissue (10% to 50%) embedded between collagen bundles of the dermis.^5^NLCS shows no preference based on gender or family history and is frequently devoid of any associated congenital defects.^1^ Treatment is not indicated except for cosmetic purposes.^4^We report a case of congenital classical NLCS in a 12-year-old boy presented with multiple sessile growths and a cerebriform surface-an uncommon condition. We intend to highlight the clinical and histopathological features, as well as the differential diagnosis of this condition.

## Case Report

A 12-year-old boy presented with a history of multiple painless lesions confined to the right gluteal region since birth. The lesions began as a single nodule and over time, new lesions developed and increased in size. The lesions were soft, skin colored and non-tender. The lesions caused no symptoms except for an unsightly appearance. There was no family history of similar lesions.

**Figure 1:**
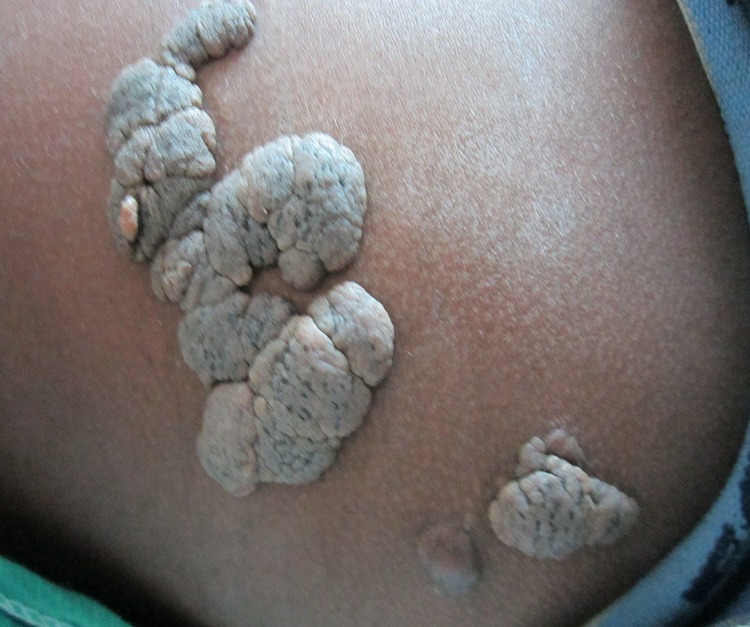
Skin-colored plaques with cerebriform surface, comedo-like plugs and nodule seen on the right gluteal region.

Physical examination revealed several skin-colored, well-defined, soft and sessile growths with a cerebriform surface with centrally located comedo-like plugs that spread over an area of approximately 12×4 cm on the right gluteal region. There was no ulceration, excessive hair growth, pigmentation, café-au-lait macules or induration. Systemic examination was unremarkable (figure 1).

Routine investigations such as hemogram, blood biochemistry that included a serum lipid profile and urine analysis were within normal limits, as follows: Hb (12.5 gm%), total leucocyte count (7800/cumm), neutrophils (72%), lymphocytes (28%), and platelet count (2.5 lakhs/cumm). Urine analysis revealed no glucose, ketone bodies, protein, or blood with a microscopy of 0-2 epithelial cells, no pus cells and no casts. Serum lipid profile results included: serum cholesterol (164 mg/dl), serum triglycerides (110 mg/dl), HDL cholesterol (92 mg/dl), and LDL cholesterol (100 mg/dl).

No oral or topical medications were prescribed for the patient. Staged excision was performed until the lesion was completely removed.

Hematoxylin and eosin (H&E) stained sections of the lesion revealed slight hyperkeratosis, papillomatosis and elongation of rete ridges of the epidermis. Ectopic adipocytes were embedded within the collagen bundles in the dermis with no connection of these adipocytes with the subcutaneous fat. The adipose tissues were not encapsulated and were mature. Dermal adnexa were reduced. 

**Figure 2:**
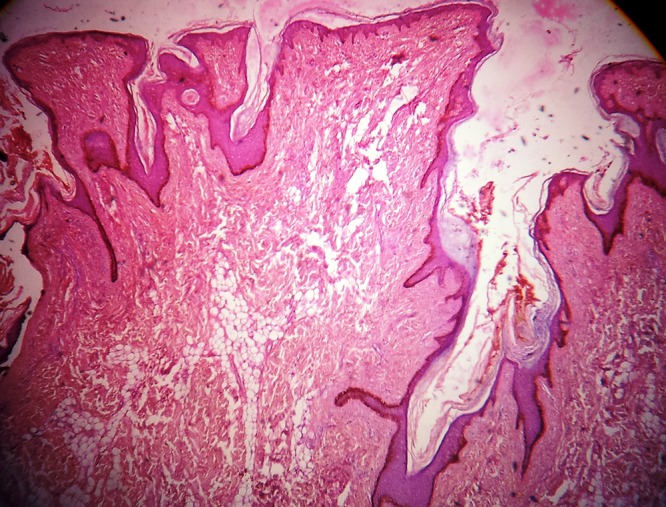
Photomicrograph of epidermal hyperkeratosis, papillomatosis, and elongation of rete ridges with mature adipose tissue in the dermis (H&E, 4×).

**Figure 3:**
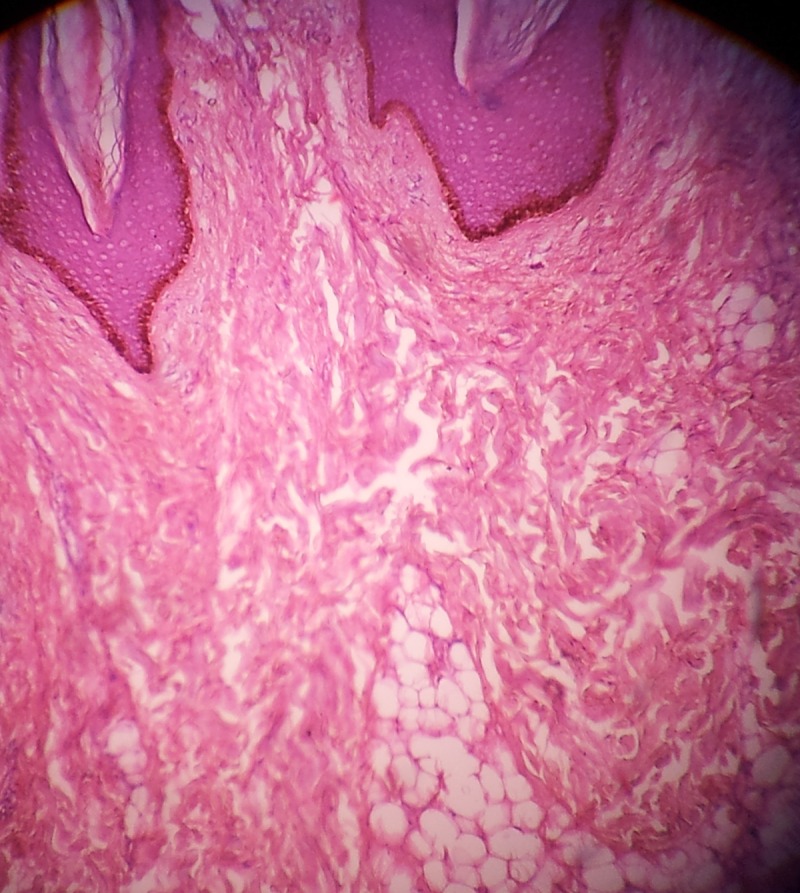
Photomicrograph showing adipose tissue embedded in the dermal collagen (H&E, 10×).

According to the histological findings, a diagnosis of NLCSof the right gluteal region was made. No recurrence was observed in the six-month follow-up (figures 2 and 3).

## Discussion

In 1921, NLCS was first described by Hoffmann and Zurhelle. It is a developmental anomaly that may be present at birth or may even begin in infancy (nevus angiolipomatosus of Howell). If the disorder begins during infancy, the change of hypoplastic dermis leads to pseudotumor yellow protrusions concurrent with skeletal and other malformations. These are usually seen during the first two decades of life, after which they become infrequent.^6^^,^^7^In the current case NLCS was present since birth.

Clinically, there are two types: classical (multiple) type and solitary.^1^^,^^4^^,^^5^^,^^8^ The classical type occurs at birth and is seen frequently in the pelvic girdle, buttocks, lower back or upper thighs. This type is characterized by plaques or cerebriform masses consisting of numerous papules and nodules distributed in zones or in a zosteriform fashion.^1^The lesions are slow-growing but can reach a large size if left untreated, as with the current case.

The second clinical form of NLCS is a solitary papule or nodule mimicking a skin tag. This type usually appears during the third to sixth decades of life and can occur anywhere on the skin.^4^ Solitary types have been noted in rare sites such as the scalp, eyelids, nose and clitoris.^9^^-^^13^ As the solitary form shows clinical and pathological features that differ from the classical type, it is also referred to as pedunculated lipofibroma.^14^^,^^15^

Family history and predominant sex involvement have not been reported in either clinical type.^1^^,^^3^^,^^4^^,^^8^

NLCS is almost always asymptomatic as noted in the current case, although rarely ulceration may occur after external trauma or ischemia.^16^Café-au-lait macules, leukodermic spots, overlying hypertrichosis and comedo-like alterations sometimes coexist.^3^^,^^4^^,^^7^ Similarly, in our case the surface of the nevus was studded with multiple open comedons. Several authors have found NLCS in the presence of other cutaneous conditions such as follicular papules and hypertrophic pilo-sebaceous lesions, angiokeratoma of Fordyce and hemangioma.^16^

Although fat deposition in the dermis has previously been considered to be a consequence of degenerative changes in connectives tissues, the pathogenesis of NLCS remains unknown. None of the studies have substantiated this theory. Presumably, fat cells in the dermis were the result of local heterotopic development of adipose tissues. NLCS was presumed to be the result of displacement of subcutaneous adipose tissues embedded into the dermis. Recently, electron microscopic findings strongly confirmed the perivascular origin of young adipocytes and the differentiation into mature fat.^3^

Some authors have classified NLCS as a connective tissue nevus based on the observed changes in mesenchymal dermal components other than fat cells, such as collagen, elastic fibers, fibroblasts and blood vessels. Cases of connective tissue hamartomas with altered epithelial elements are rare. Recently, a report of a NLCS with a 2p24 deletion has been published. The role of genetic abnormalities in the development of NLCS is inconclusive; therefore, further studies are needed for confirmation and clarification of a possible relationship between NLCS andconnective tissue nevus.^1^

Histologically, collagen bundles of the dermis show fat cells that have frequent extension to the papillary layer. In instances with relatively large amounts of fat, fat lobules are irregularly distributed throughout the dermis and the boundary between the dermis and hypodermis is ill-defined or lost. The fat may all be mature, but in some instances an occasional small, incompletely lipidized cell may be observed. In cases with only small deposits, the fat cells are apt to be situated in small foci around subpapillary vessels.^7^

Usually the dermis is normal even with the fat cells but in some cases of NLCS there is an increase in the density of collagen bundles, fibroblasts and blood vessels in the dermis.^7^

Epidermal findings such as “mild to moderate acanthosis, basket weave hyperkeratosis, increased basal pigmentation and focal elongation of rete ridges” have been noted.^17^In many instances although the number of adnexal structures is reduced in NLCS compared to normal adjacent skin their morphology remains unaltered. Several studies have documented cases of NLCS with pilar anomalies such as abortive hair germ like structures, hypertrophic pilosebaceous units, perifollicular fibrosis, and folliculosebaceous cystic hamartomas.^1^

NLCS should be clinically differentiated from nevus sebaceous, neurofibroma, lymphangioma, focal dermal hypoplasia, cylindroma, trichoepithelioma, and angiolipoma. Histopathological evaluation is required for diagnosis and is based on the presence of ectopic mature adipocytes that proliferate in the reticular dermis with possible extension to the papillary dermis and intermingled with collagen bundles.^16^Although the usual absence of connection to subcutaneous fat tissue is most characteristic of NLCS, some authors use it as a ‘necessary criterion for diagnosis.^18^

Intradermal melanocytic nevus and Goltz syndrome show histopathological pictures similar to that of NLCS, however they can be readily differentiated from NLCS based on clinical features.^6^ NLCS should be differentiated from focal dermal hyperplasia which in addition to clusters of adipocytes in the dermis, there is extensive attenuation of collagen.^7^

For cosmetic purposes, surgical excision is the best choice of treatment. If left untreated they can eventually increase in size causing apprehension and cosmetic concern. Malignant degeneration and recurrences are extremely rare and to the best of our knowledge have not been reported.

## Conclusion

This rare case of congenital classical NLCS presented as cerebriform lesions with centrally located comedo-like plugs. Though not known for malignant degeneration, physicians should be aware of this distinct condition for early intervention, as it can grow to a large size causing apprehension for the patient.
